# PT symmetry enforced twin exchange as the origin of chirality-induced spin selectivity

**DOI:** 10.1126/sciadv.aec7069

**Published:** 2026-03-18

**Authors:** Pius M. Theiler, Sander Driessen, Matthew C. Beard

**Affiliations:** ^1^ National Laboratory of the Rockies, Golden, CO, USA.; ^2^ Department of Chemistry, Duke University, Durham, NC, USA.; ^3^ Department of Applied Physics, Eindhoven University of Technology, Eindhoven 5612 AZ, Netherlands.

## Abstract

Chiral molecules, ubiquitous in chemistry and biology, can differentiate electrons by their spin, a phenomenon known as chirality-induced spin selectivity (CISS). Despite its robustness and technological relevance, CISS has resisted conventional explanation: Spin-orbit coupling (SOC) models cannot fully account for the observed magnitude, room-temperature persistence, or equilibrium signatures. Here, we argue that structural chirality enforces a twin-pair exchange mechanism via the indistinguishability principle, which intrinsically couples spin and spatial degrees of freedom such that wave functions cannot be factorized into spin and spatial components. We derive an effective Hamiltonian that describes both transport and equilibrium CISS phenomena and is non-Hermitian. However, the inherent pseudo-Hermiticity, with PT symmetry as a special case, ensures real eigenvalues and thermodynamic consistency. We demonstrate that our framework is a step toward resolving long-standing anomalies of CISS. It situates CISS alongside equilibrium symmetry-breaking phenomena such as ferromagnetism and superconductivity, with implications for spintronics, catalysis, and the origins of biological homochirality.

## Teaser

Electron correlations in chiral systems drive PT symmetry and the emergence of spin-selective transport.

## INTRODUCTION

Chirality-induced spin selectivity (CISS) is notable in both magnitude and robustness (*1*–*3*): It persists at room temperature, appears in closed-shell molecules, and manifests across transport, photoemission, and catalytic platforms. However, the traditional spin-orbit coupling (SOC) along with decoherence (*4*) or dissipative influences (*5*), while conceptually intriguing, can only partially account for many of the observed CISS phenomena. Theoretical efforts (*3*) have focused on dynamic, dissipative, phonon-mediated scenarios: spin-orbit-coupled transport, nonequilibrium Green functions, or Lindblad dynamics (*6*–*9*). These single-particle approaches have difficulties to account for zero-bias magnetoresistance (*10*, *11*), magnetization (*12*), and work-function shifts (*13*, *14*) observed in equilibrium. They imply dissipative dynamics where some experiments (*12*, *15*) instead reveal persistent, time-independent signals. Recent ultrafast inverse CISS (*16*), magnetoresistance measurements (*10*, *11*), and unusual electron pairing (*17*) sharpen this paradox. Some of these aspects of CISS have previously been attributed to correlation effects (*18*, *19*), including driven models in which charge-charge electronic correlations generate effective spin polarization (*20*–*22*). These approaches have provided important insight into how correlations can couple chirality to spin-dependent observables. However, the connection to genuine equilibrium properties remains subtle as driven, correlation-induced dynamics can be formally related to closed, highly correlated systems (*23*). In this work, we adopt a complementary, explicitly equilibrium perspective: a closed, correlated system in which the apparent non-Hermiticity arises from integrating out many-body degrees of freedom, yielding an effective single-particle projection. Within this picture, chirality imprints equilibrium correlations that extend beyond traditional SOC-based mechanisms.

**Fig. 1.**
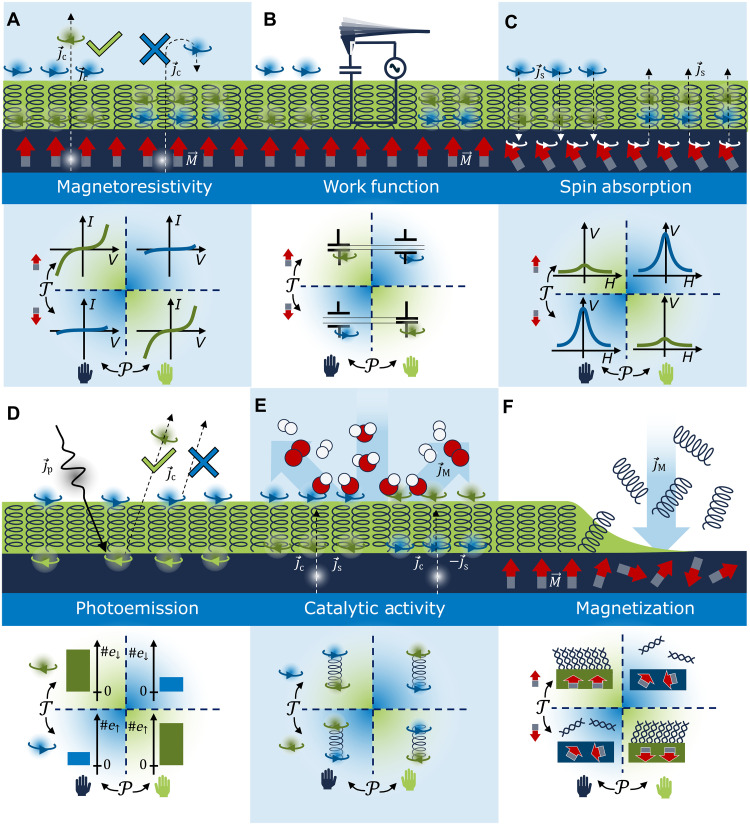
Representative classes of CISS experiments, each exhibiting broken P and T symmetries but preserved PT invariance. (**A**) Magnetoresistance measurements show distinct current-voltage relation (*I*-*V*) characteristics depending on the enantiomer and the magnetization direction $\vec{M}$ of the ferromagnetic substrate. (**B**) Quantum capacitance and contact potential difference measurements using Kelvin probe force microscopy reveal different spin and charge distributions for opposite enantiomers. (**C**) In spin-pumping experiments, a spin current ${\vec{j}}_{s}$ detects spin-to-charge conversion as an inverse spin Hall voltage V or changes in absorption spectra upon switching either magnetization or molecular chirality. (**D**) In photoemission, the photon current ${\vec{j}}_{p}$ ejects spin-polarized electrons with spin-resolved yields #$e_{↑}$ and #$e_{↓}$ that dependent on the stereochemical configuration. (**E**) Spin-selective catalysis produces spin-polarized charge currents ${\vec{j}}_{c}$. (**F**) Adsorption of chiral molecules induces a net magnetization in initially nonmagnetic substrates. In all cases, experimental outcomes change upon flipping either the magnetization or the enantiomer but remain invariant under the combined PT operation, highlighting the shared symmetry structure across CISS phenomena.

At the heart of this puzzle lies a symmetry structure. Experiments consistently show that CISS observables break both parity (P) and time-reversal (T) symmetries, while preserving their product, PT (Fig. 1). This pattern is observed in magnetoresistance (*10*, *11*), quantum capacitance (*15*), spin-to-charge conversion (*16*, *24*–*26*), photoemission (*14*, *27*, *28*), spin-selective catalysis (*29*–*34*), and interface magnetization (*12*, *35*–*37*): Flipping either the stereochemical configuration or spin orientation reverses the signal, but flipping both restores it. Such behavior cannot be captured within a conventional Hermitian inner product (*38*–*41*).

We propose a conceptual shift. Exchange symmetry, a cornerstone of quantum mechanics, acquires a previously unidentified role in nonlocal chiral systems: It enforces a twin-pair exchange mechanism that couples spin and spatial degrees of freedom. In these states, the wave function cannot be factorized into spatial and spin parts $\Psi \left(x_{i},\sigma_{i}\right)\neq \phi \left(x_{i}\right)\;\varphi \left(\sigma_{i}\right)$ (*42*).

The effective, free Hamiltonian is PT-symmetric as a special case of a more general pseudo-Hermitian structure (*41*) and connected quantum metric (*43*), ensuring real spectra and unitary time evolution. Thus, we find that Hermiticity with respect to the conventional inner product is not a fundamental physical requirement, but a convenient mathematical choice for thermodynamic consistency (*44*). Our PT chiral spin exchange framework captures the equilibrium nature of CISS and distinguishes it fundamentally from traditional local SOC-driven dynamics (*3*).

PT symmetry is well established (*45*), especially in photonic systems, where gain-loss balance yields non-Hermitian Hamiltonians with topologically protected modes and exceptional points (*46*, *47*). Here, a pseudo-Hermitian symmetry, realized here in a PT-symmetric form, emerges in a closed, correlated electronic system, where the effective non-Hermiticity originates from exchange interactions rather than external reservoirs. Our focus is on the microscopic origin and consequences of this correlation-driven PT symmetry for the description of CISS. Our formulation further connects to known non-Hermitian phenomena: The twin exchange drives a chiral analogue of the non-Hermitian skin effect (*48*, *49*), producing spin accumulation and charge separation. By grounding CISS in PT-symmetric exchange physics, we provide a unifying principle that rationalizes its diverse manifestations and situates it alongside thermodynamical equilibrium phases that are Tsymmetry breaking such as ferromagnetism and superconductivity.

## RESULTS

Structural chirality is defined by the absence of any mirror symmetry, and, in quantum systems, it is not a property of individual particles alone but of their collective arrangement. Detecting chirality requires comparing spatial relations across multiple particles, a fundamentally nonlocal operation (*50*, *51*). To encode this information, we introduce an internal index ξ that labels the global stereochemical configuration of the multielectron wave function. Although ξ is inaccessible to local probes, it governs how the state transforms under particle exchange.

**Fig. 2.**
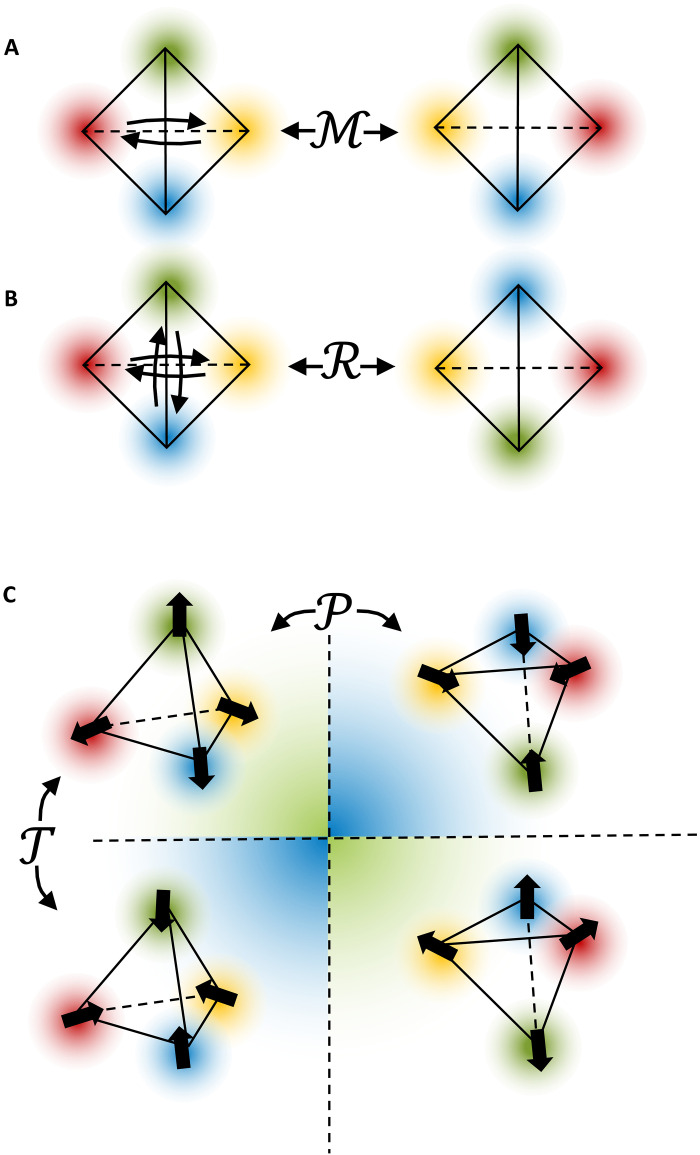
Chiral symmetry and twin exchange in a tetrahedral multielectron system. (**A**) A single pairwise exchange of particles in the chiral tetrahedron transforms the system into its mirror image (M), effectively acting as a combined parity and rotation operation that reverses the stereochemical configuration. (**B**) A coordinated twin exchange where two disjoint correlated pairwise swaps rotates (R) the tetrahedron while preserving its handedness. (**C**) Visualization of the spin and charge densities for four distinct, color-coded multielectron wave functions $\Psi^{\xi }$ encoding nonlocal stereochemical information. Charge densities are shown as shaded vertex clouds; spin textures are indicated by vector arrows. The schematic highlights how chirality intrinsically breaks parity (P) and time-reversal (T) symmetries while preserving their combination (PT).

In conventional systems, the spin-statistics theorem dictates that the exchange of two indistinguishable fermions multiplies the wave function by −1 (*52*). In chiral many-body systems, the chirality of a multielectron configuration can be determined from the permutation parity (even or odd) of the ξ indices relative to a reference ordering of the nuclei (Fig. 2A). A single-electron exchange corresponds to an odd permutation of the ξ indices, which flips the parity and thus changes the chirality of the wave function relative to the fixed nuclear framework. This makes the resulting configuration physically distinguishable, violating the indistinguishability principle at the core of exchange symmetry. The resolution is a twin exchange: a correlated operation involving two disjoint pairwise swaps, which corresponds to an even permutation of the ξ indices and therefore preserves the overall chirality of the multielectron state (Fig. 2B). These generalized exchanges of particles between spin-space coordinates $x_{j}↔x_{j+1}$ act not as simple scalar phases but as matrices $R_{\chi }$ preserving the parity of ξ if properly chosen (see the “Group theoretical analysis” section), Wang and Hazzard (*53*) extended the spin-statistics theorem to nonlocal systems

$${\Psi^{\xi }\left.\left({\left\{x_{i}\right\}}_{i=1}^{4}\right)\right|}_{x_{j}↔x_{j+1}}=\sum_{J}{\left(R_{\chi }\right)}_{J}^{\xi }\;\Psi^{J}\left({\left\{x_{i}\right\}}_{i=1}^{4}\right)$$ (1)

where $R_{\chi }$ are exchange matrices acting on the stereochemical Hilbert space, satisfying $R_{\chi }^{2}=𝕀$ and $\left[R_{j},R_{k}\right]=0$.

The algebra of such twin exchanges is non-Abelian when projected onto single-particle degrees of freedom (*54*), so that it generates effective non-Hermitian terms (see the “Nonlocal kernel” section). The simplest consistent form arises in a Dirac description (*55*, *56*), where the spin and space degrees of freedom can be treated on equal footing: Summing the symmetry allowed (chirality preserving) correlated exchanges introduces a pseudoscalar contribution proportional to $\mathrm{im}{\alpha \gamma }^{5}$, where α=K/mc and K is the energy cost of the microscopic twin exchange. The inclusion of the symmetry allowed twin exchanges naturally leads to Dirac’s fifth matrix, $\gamma^{5}$, whose eigenstates correspond to definite states of chirality, ±1. Thus, $\gamma^{5}$ reflects the sensitivity to global stereochemical configuration. Although Dirac made use of the Lorentz-invariant Clifford algebra, omitting the $\gamma^{5}$ matrix in his original derivation (*55*), it has been shown that Dirac’s theory can be generalized to include $\gamma^{5}$ (*56*). Taking the nonrelativistic limit yields an effective single-particle Schrödinger equation (see the “Nonrelativistic limit of Dirac equation” section)

$$\left(\frac{{\hat{p}}^{2}}{2m}+{\hat{V}}_{0}-i\alpha \;\hat{\sigma }\cdot \hat{p}\right)\Psi =E\Psi$$ (2)

where α is a velocity scale set directly by the twin-exchange strength accounting for the degree of spin-momentum locking and thus for CISS. Physically, α plays the role of a chiral drift speed analogous to the Rashba velocity in traditional SOC systems, with energy scale of $\frac{1}{2}\alpha^{2}m$, but, here, it originates from exchange statistics rather than single-particle relativistic SOC. The emergent term $i\hat{\sigma }\cdot \hat{p}$ explicitly breaks the parity (P) and time-reversal (T) symmetries individually while preserving their combination. Thus, the generalized exchange framework produces a non-Hermitian Hamiltonian. In this work, we restrict attention to the free Hamiltonian with constant potentials, for which PT symmetry ensures thermodynamic consistency (*57*). A more general formulation accommodating arbitrary spatial potentials is developed in (*43*).

This behavior is illustrated in Fig. 2C: Spins in opposite stereochemical configurations align either inward or outward, preserving a net-zero magnetic moment. T inverts the spin configuration; P inverts the stereochemical configuration; only the combined PT operation leaves the electronic wave function invariant and thus preserves indistinguishability. This is the defining symmetry of the chiral spin interaction. Any other spin configuration would generate a net magnetic moment, violating experimental constraints and PT symmetry.

If the only allowed symmetry operations are twin exchanges that preserve ξ, then the elementary fluctuation is not a sequence of two independent pair swaps. Rather, it corresponds to a single, nonlocal, correlated flip of two disjoint pairs. Therefore, it is sufficient to consider the transition of a single pair exchange when estimating the velocity scale α because the second flip is enforced by symmetry. Consequently, the estimate for the energy scale $\frac{1}{2}\alpha^{2}m$ is of the order of exchange interactions, i.e., K of tens to hundreds milli–electron volts, yielding $\alpha \approx 10^{4}\;\text{to}\;10^{6}$ m/s.

To explore the physical consequences of the non-Hermitian Schrödinger equation derived above, we consider a one-dimensional (1D) model with hard-wall boundary conditions. This reduction is justified because the Hamiltonian is separable in the spatial coordinates (*39*, *40*), so the dynamics along each orthogonal axis can be treated independently. When discretized (see the “Analytical 1D model derivations” section), the resulting 1D non-Hermitian Hamiltonian exhibits asymmetric hopping terms for spin-up and spin-down electrons. Each spin component is described by a Hatano-Nelson–type model (*58*), leading to eigenstates that are real and doubly degenerate but spatially localized at opposite ends of the system depending on spin orientation. Furthermore, the twin-exchange correlation leads to a shift of the spectra by $\frac{1}{2}m\alpha^{2}$ compared to the nonchiral solution. This framework allows us to study the non-Hermitian skin effect and spin-dependent localization in a controlled, analytically tractable setting.

**Fig. 3.**
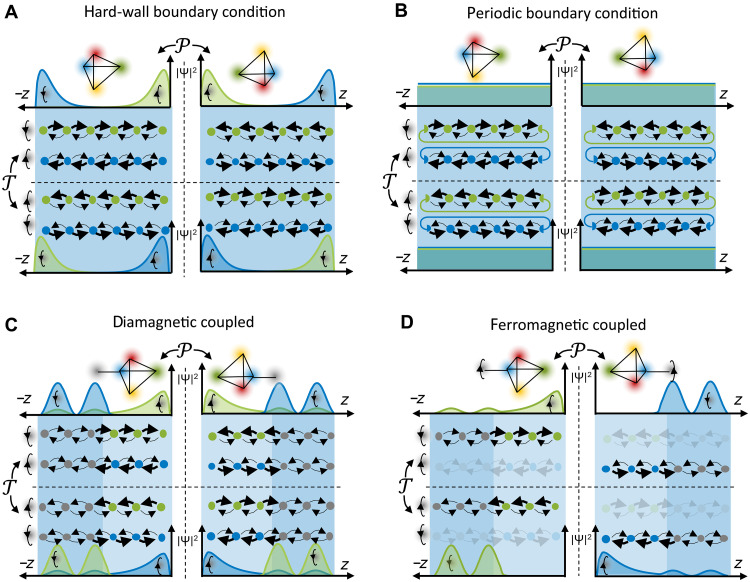
Non-Hermitian spin localization in chiral systems under different boundary conditions and leads. (**A**) Under hard-wall boundary conditions, spin-up and spin-down states localize at opposite interfaces due to asymmetric hopping. (**B**) With periodic boundary conditions, the states delocalize across the system, eliminating the skin effect. (**C**) When a chiral domain is coupled to a diamagnetic conductor, spin-selective accumulation or depletion occurs at the interface, with no net magnetization. (**D**) Coupling to a ferromagnetic material introduces spin-dependent energy shifts, favoring one spin species and producing two distinct energy states: one extended and one confined. In all panels, PT-conjugate states appear as symmetry-related pairs across diagonals, reflecting the preserved PT symmetry of the Hamiltonian.

Figure 3A shows the probability densities of the resulting states. Spin-up (green shading) and spin-down (blue shading) wave functions accumulate at opposite interfaces, realizing a chiral spin texture consistent with the PT-symmetric configuration shown in Fig. 2C. Under P (left to right), the localization flips spatially; under T (top to bottom), the spin flips. Only their combination leaves the full state invariant (evidenced by the diagonal configurations) reinforcing the emergent symmetry of the chiral spin state.

The spectrum remains real as long as the coupling strength satisfies ∣α∣<ℏ/(mΔz), a hallmark of PT-symmetric systems (*44*). The structure of the solid-state system itself enforces this bound: α cannot exceed ℏ/(mΔz) because of the finite spatial extent Δz of the electron wave function. In other words, the system is intrinsically constrained to remain in the PT-unbroken regime. This limiting value can be interpreted as a maximum effective velocity set by the chiral enforced electron correlations.

When periodic boundary conditions are imposed, the spin-resolved localization vanishes and states become fully delocalized, as shown in Fig. 3B. This illustrates the non-Hermitian skin effect: Wave functions localize at boundaries in finite systems, but not in the periodic case violating the usual bulk-boundary correspondence of Hermitian systems (*48*).

## DISCUSSION

The above analysis identifies two intrinsic consequences of the chiral twin-exchange electron correlation that are independent of microscopic material details. First, the non-Hermitian exchange term induces a spin-resolved localization when the system is terminated, producing a boundary magnetization that is opposite but of equal magnitude at the two ends. Second, the spin-momentum correlation generates a characteristic kinetic-energy rearrangement of order $\frac{1}{2}\alpha^{2}m$, which sets the effective exchange scale of the system. Crucially, both effects are properties of the isolated system under equilibrium conditions, not a dynamical nonequilibrium response. In our framework, we find that most, if not all, CISS experiments can be interpreted as boundary condition–dependent probes, each revealing aspects of the same two equilibrium features of the chiral interface. We discuss how a wide range of experimental observations can be understood by analyzing how the two fundamental ingredients manifest under different boundary conditions and how specific measurements probe/access the resulting boundary magnetization.

To make this connection explicit, we organize the discussion around the four boundary conditions shown in Fig. 3: (A) an isolated chiral system that is terminated on both sides by an insulator (e.g., vacuum, solvent, or dielectric), (B) a periodic chiral system with no boundary termination, (C) a chiral layer coupled to a diamagnetic conductive substrate (e.g., a chiral/normal metal contact), and (D) a chiral layer coupled to a ferromagnet. The resulting spin localization and the associated energy shifts can then be accessed either in quasi-equilibrium measurements, i.e., probing the local magnetization or voltage differences, or in transport measurements probing current and optical experiments, probed via charge ${\vec{j}}_{c}$, spin ${\vec{j}}_{s}$, or photon currents ${\vec{j}}_{p}$ illustrated in Fig. 1.

### Isolated chiral system

Figure 3A illustrates that, in an isolated chiral system with hard-wall boundary conditions, spin-up and spin-down electrons localize at opposite ends of the domain, generating a boundary-localized spin polarization that is equal in magnitude but has opposite orientation at either boundary. Symmetry protects spin polarization: Inverting the enantiomer flips the spatial spin distribution without changing the overall effect that has been experimentally observed (*59*), and the system remains in equilibrium without dissipation or external bias.

Experimentally, the configuration is analogous to a chiral system in contact with a solvent and integrated in an electrocatalysis setting. The solvent provides the electrostatic barrier which imposes the hard-wall boundary conditions to the chiral system. The boundary spin polarizations act as spin reservoirs that can selectively transfer angular momentum to surface bound reactants while preserving the PT symmetry of the system under current perturbation. Spin-displacement enhances the performance of chiral electrocatalysis in reactions involving spin-dependent reactants, notably molecular oxygen, which has a triplet ground state (*29*). In such reactions, spin-polarized electrons promote the formation of spin-matched reaction intermediates (*29*), thereby lowering the activation barrier for the desired product (*31*) or enabling the formation of enantiopure stereocenters (*33*).

For instance, it has been shown that spin-polarized currents can enhance the oxygen evolution reaction (*29*, *34*). These results are puzzling within the framework of conventional spintronics, as they arise even in the absence of ferromagnetic electrodes or strong SOC. Furthermore, Liang *et al.* (*32*) observed that catalytic activity remains enantioselective even without external current applied through the chiral material. Such observations are suggestive of and compatible with theoretical models in which a persistent equilibrium spin texture contributes alongside electrostatic and steric interactions, and such phenomena is captured in our simple model. Because the chiral interface spatially separates the spin species, it provides a more effective spin bias than ferromagnets, where minority and majority carriers spatially coexist.

The enhancement of reactions involving triplet intermediates results from statistical matching between the interfacial spin polarization and the spin state of the reactants (*30*). In this sense, an enantiopure chiral material provides a highly spin-polarized interface that promotes the formation of spin-allowed intermediates.

In addition to favoring reactions involving spin-dependent intermediates, our model also offers insight into the formation of enantiopure stereocenters. The interface-localized spin states can act as a template that preferentially stabilizes one stereoisomer over the other. Providing predominantly a single spin species at the reaction site may prime one side of the molecule, thereby biasing the reaction pathway toward a specific handedness, consistent with recent reports on spin-polarized induction of enantioselectivity (*33*, *60*). In systems without steric constraints, such a spin-dependent bias could become the dominant contribution to enantioselective product formation.

Experiments on donor-chiral-acceptor molecules can likewise be interpreted within the framework of isolated terminated systems, demonstrating that a metal/chiral interface is not required to observe CISS effects (*61*). In this probing geometry, a static magnetic field lifts the spin degeneracy, and time-resolved electron paramagnetic resonance detects the resulting spin-selective excited-state dynamics. Because optical excitation couples directly with the electronic momentum and the chiral Hamiltonian correlates the momentum with spin, the excitation will also reflect that spin-momentum correlation. While this interpretation is consistent with the symmetry structure of the presented model, determining the precise dynamics will require further detailed investigation.

### Infinite, periodic systems

Figure 3B illustrates that, under periodic boundary conditions, the wave functions become fully delocalized. In contrast to terminated systems, no energy splitting or boundary-localized spin polarization appears. Consequently, CISS-related signatures are not expected in perfectly periodic infinitely extended systems.

To our knowledge, this boundary condition–driven localization-delocalization transition has not yet been directly tested experimentally. Several experimental platforms offer opportunities: programmable metamaterials (*17*), where closing a linear geometry into a ring could reveal changes in conductivity; chiral porphyrin nanobelts versus open ribbons (*62*), where nearby nuclear magnetic resonance-active probes could detect boundary spin accumulation; and trapped ions arranged in chiral lattices (*63*), where individual states can be read out with high fidelity. Because such effects do not arise in Hermitian models (*48*), observing them would provide a decisive test of the present mechanism.

### Diamagnetic coupled system

Figure 3C illustrates a chiral system coupled to a diamagnetic system, e.g., a chiral systems in contact with a metal. As an extension of the isolated system, the spin accumulation at the chiral interface does not remain confined to the chiral domain itself; it can also magnetize adjacent diamagnetic or paramagnetic materials, so that they show localized, finite magnetization at that interface.

If chiral/diamagnetic heterostructures are probed experimentally with a pure charge current, then the spin-momentum correlations impose the generation of an accompanying colinear spin current. In spin light-emitting diodes, such spin current generation has been demonstrated using a chiral perovskite in contact with a light-emitting quantum well structure (*64*). The generation of circular polarized electroluminescent light is direct indication of the induced spin polarization even in the absence of ferromagnetic contact.

In photoemission experiments (*28*, *65*), the photoelectrons originate from the metallic substrate that is in contact with the chiral system. The spin polarization is detected at a Mott detector providing a direct probe of the interfacial spin population at the moment of emission (*66*). In contrast to a transport-based interpretation where unpolarized spins are spin filtered through the chiral molecular overlayer, our framework attributes the observed photoemission asymmetry primarily to the initial interfacial spin population. The chiral overlayer only minimally affects the photoelectron propagation. Instead, the chiral layer modifies the local density of states and establishes a spin-dependent interfacial polarization, which biases the emission of spin-up and spin-down electrons. Consequently, stronger or thicker chiral layers can enhance the interfacial spin imbalance and lead to a larger detected spin polarization. This increase saturates once the thickness exceeds the interfacial screening length because additional material no longer contributes to the local polarization. Spin-dependent photoelectron yield associated with opposite light helicities can superpose with this intrinsic CISS-induced emission imbalance but do not constitute its origin.

### Ferromagnetic coupled system

In the ferromagnet-chiral composite (Fig. 3D), the degree of spin-selective wave function localization at the interface depends on the compatibility between the enantiomeric spin configuration and the magnetic polarity of the ferromagnet. Both ingredients of the model, i.e., the spin-selective boundary localization and the kinetic-energy shift $\frac{1}{2}\alpha^{2}m$ contribute to the interfacial energetics. When the preferred spin orientation of the chiral domain matches the magnetization of the ferromagnet, the non-Hermitian skin effect enhances the wave function overlap with the interface, increasing screening, stabilizing the local magnetization, and lowering the work function, consistent with models (*22*). In the opposite configuration, the wave function is repelled, reducing screening and increasing the work function. A simple electrostatic band-bending model (*15*) links these changes to variations in quantum capacitance at thermodynamic equilibrium.

Experimentally, the coupling between ferromagnets and chiral layers is well established. The chiral layers adsorbed onto small (super)paramagnetic nanoparticles were shown to induce robust ferromagnetic behavior (*67*). In other experiments, a chiral layer is placed in contact with a ferrometallic electrode, and changes in magnetization or coercive field are recorded as a function of enantiomer type (*12*, *68*). Dor *et al.* (*12*) showed that adsorption of enantiopure molecules on a ferromagnet with perpendicular anisotropy can reverse its magnetization without external magnetic fields. More recently, Meirzada *et al.* (*68*) used nitrogen-vacancy magnetometry to monitor the vectorial magnetization reorientation and correlated it with the gradual change in the molecular tilt angle. Their data indicate that the magnetization reversal is a long-lived, equilibrium process stabilized by exchange interactions mediated through the spin-polarized boundary states of the chiral layer.

These persistent effects also manifest electrostatically. Shifts in work function (*13*–*15*) and changes in quantum capacitance (*15*) have been directly observed at chiral-ferromagnet interfaces. Using Kelvin probe force microscopy, Theiler *et al.* (*15*) resolved differences in contact potential and quantum capacitance for opposite enantiomers and opposite magnetization orientations. A time-resolved measurement indicated that these shifts are time independent and did not depend on the perturbation applied by the atomic force microscopy tip, demonstrating that the time-reversal breaking is intrinsic to the system and not imposed by the probing strategy. While adsorption of molecular overlayers generally induces electrostatic work-function shifts on both magnetic and nonmagnetic substrates, the magnetization- and enantiomer-dependent shifts discussed here are experimentally observed only for chiral layers coupled to ferromagnetic order.

When a charge current is used as the probe, these interfacial energetics manifest as magnetoresistance. Because magnetoresistance reflects only the charge-transport channel, any observed chirality-dependent magnetoresistance must therefore originate from the charge sector. Tirion and van Wees (*69*), theoretically extending the quantum-capacitance model (*15*), attributed the magnetoresistance to changes in the effective barrier height at the chiral-ferromagnet junction. Their model captures the experimental features but required an unexplained enantiomer- and magnetization-dependent work-function shift. The missing microscopic ingredient is provided by our PT model, which predicts spin-selective charge localization that induces an electrostatic modulation of the barrier through non-Hermitian boundary conditions. The barrier itself remains non–spin selective and only modulates the charge current.

This interpretation also resolves one of the most puzzling observations in CISS: chirality-dependent magnetoresistance at equilibrium, without any current through the chiral layer (*10*, *11*). The resistance of a chiral-ferromagnet junction depends on both the magnetization direction and the handedness even at zero bias. This behavior is incompatible with conventional transport mechanisms but consistent with an equilibrium shift of the interfacial energetics induced by the chiral skin effect. Further support comes from the tunneling model (*11*), which fits current-voltage relation (*I-V*) curves by invoking a magnetization- and enantiomer-dependent barrier height. The physical origin of this barrier modulation, however, becomes clear only in the present framework: Spin-selective localization modifies the local density of states and the electrostatic potential landscape, thereby modulating the tunneling barrier. Photoemission measurements of similar ferromagnetic-chiral composite junctions (*14*) provide an additional probe of differences in work function under ambient conditions.

Last, probing the composite with a pure spin current reveals the inverse effect. The non-Hermitian coupling term $i\alpha \;\hat{p}\cdot \hat{\sigma }$ enforces spin-momentum locking at equilibrium. In steady state, spin and charge densities arrange so that interfacial localization remains balanced. Injecting a spin current perturbs this balance, forcing a compensating charge flow to restore the intrinsic spin-charge correlation, thereby generating an electrical signal. This inverse CISS effect has been observed experimentally through spin pumping into chiral layers (*16*, *24*–*26*). The picosecond-scale response measured by Dong *et al.* (*16*) and the correlated spin-momentum signatures reported by Moharana *et al.* (*24*) highlight the correlative electronic origin of the effect. This supports an interpretation in which the notion of local equilibrium governs the interfacial dynamics.

### Rethinking CISS

This framework bridges two long-standing debates: whether CISS is a bulk or interface effect and whether it requires out-of-equilibrium conditions. In our model, both bulk symmetry breaking and boundary-induced localization are essential. From this perspective, spin selectivity is an equilibrium property emerging from the interplay of exchange interactions and geometry. While our analysis begins from the simplest case, rather than a full ab initio treatment of molecular systems, it takes on the conceptual step that spin statistics can be generalized to nonlocal settings (*53*). This insight reframes how we think about spin transport in molecular systems, and why existing simulation tools, built on Hermitian and periodic assumptions, often fail to capture CISS. By highlighting exchange correlations as the key driver, the framework opens an unexplored direction for calculating spin-selective effects with methods developed in the context of superconductivity, now applied to structurally chiral quantum systems.

Several predictions are directly testable. The reversal of spin accumulation under enantiomer switching, the collapse of localization under periodic boundary conditions, and the emergence of energy splitting in spin-filtered junctions all offer measurable signatures. Moreover, synthetic testbeds, such as programmable electrostatic metamaterials (*17*), nuclear magnetic resonance activity of nanobelts versus rings of chiral porphyrins (*62*), or ultracold trapped ions arranged in chiral geometries (*63*), can emulate these effects, enabling direct control of structural chiral geometry to test whether spin localization collapses under ring closure.

This work redefines chirality not as a passive geometric label but as an active quantum principle that generates a pseudo-Hermitian topology. By formulating CISS as an equilibrium phenomenon rooted in symmetry and exchange, it resolves key experimental puzzles beyond spin-orbit models (*3*) and unifies transport and equilibrium observations. More broadly, it establishes a framework on which future ab initio and molecular studies can build, linking quantum statistics, many-body symmetry, and geometry. In doing so, it sets the stage for spintronics that operate at room temperature and across disciplines, from condensed matter physics to chemistry and biology.

## MATERIALS AND METHODS

### Group theoretical analysis

Indistinguishability is constrained by the spin-statistics theorem: Exchanging two identical fermions introduces a global phase shift of π in the wave function (*52*), underpinning foundational principles such as Pauli exclusion principle and magnetic ordering (*70*). For a many-body wave function $\psi \left(x_{1},x_{2},\ldots ,x_{n}\right)$, where each particle coordinate represents the spatial and spin components $x_{j}=\left({\vec{r}}_{j},{\hat{\sigma }}_{j}\right)$, the exchange of particles 1 and 2 yields

$$\psi \left(x_{1},x_{2},\ldots ,x_{n}\right)=r\;\psi \left(x_{2},x_{1},\ldots ,x_{n}\right)$$ (3)

$$=r^{2}\;\psi \left(x_{1},x_{2},\ldots ,x_{n}\right)$$ (4)

which implies r=±1.

Applying this framework to chiral systems reveals a contradiction as explained in the main text. To restore indistinguishability, we introduce the concept of twin exchange: a composite operation involving two disjoint pairwise exchanges that preserves global chirality. These many-body correlations extend naturally to molecules with multiple stereogenic centers or chiral axes (*71*). This hints at the well-known fact that spin and charge densities transform under different symmetry constraints (*42*, *70*): While charge follows conventional point group symmetries SO(3), spin textures are governed by spin point group transformations SU(2). This allows for nontrivial spin arrangements even in the absence of SOC (*42*), a key feature of CISS phenomena.

We formalize this idea by incorporating the fundamental properties of nonlocal chirality and spin statistics, and we consider a system of n=4 electrons, each described by both a spatial coordinate and an intrinsic space-time direction representing its spin degree of freedom, $x_{i}=\left({\vec{r}}_{i},\gamma^{i}\right)$. Here, $\gamma^{i}$ is a fixed Dirac gamma matrix (*55*) encoding the spinor direction of the ith particle. This formulation treats spin and spatial degrees of freedom on equal footing, which is essential for addressing nonlocal correlations induced by structural chirality beyond Pauli-matrix algebra (*72*).

For nonlocal observables, such as chirality, the conventional spin-statistics theorem can be generalized following Wang and Hazzard (*53*). The exchange of two adjacent particles acts not only on spatial and spin degrees of freedom but also on the nonlocal stereochemical index ξ

$${\Psi^{\xi }\left.\left({\left\{x_{i}\right\}}_{i=1}^{4}\right)\right|}_{x_{j}↔x_{j+1}}=\sum_{J}{\left(R_{\chi }\right)}_{J}^{\xi }\;\Psi^{J}\left({\left\{x_{i}\right\}}_{i=1}^{4}\right)$$ (5)

where $R_{\chi }$ are exchange matrices acting on the stereochemical Hilbert space, satisfying $R_{\chi }^{2}=I$ and $\left[R_{j},R_{k}\right]=0$. The index ξ encodes the global chiral configuration and cannot be determined by any local probe. Through this nonlocal index, the phase factor r in Eq. 4 is promoted to a matrix $R_{\chi }$, capturing the correlated action of exchanges on the chiral state and providing many more possibilities (indexed with χ) than the simple r=±1 phase factor of the purely local case.

In a chiral system, the allowed $R_{\chi }$ matrices representing exchanges must be carefully constrained. Specifically, only those permutations that preserve the stereochemical configuration and do not induce physically distinguishable effects on the wave function are permitted. To construct the allowed $R_{\chi }$ matrices, we can analyze the symmetries of a chiral tetrahedral configuration (Fig. 2, A and B). The chiral tetrahedron is the simplest structure without mirror symmetry; fewer than four point-like particles always define a mirror plane. In the chiral tetrahedron, the $R_{\chi }$ matrices are restricted to a proper subgroup of the full permutation group, namely, those that square to the identity and commute with each other, ensuring consistency with both indistinguishability and the chiral nature of the system. The full set of vertex permutations forms the symmetric group $S_{4}$, containing 4!=24 elements. This is the usual outcome in the case that one assumes that the wave function factorizes into spatial and spin parts and using a simple slater determinant to antisymmetrize. However, only the 12 even permutations that preserve the handedness of the tetrahedron form the alternating group $A_{4}$ (*71*). Among these, four elements represent pure π rotations (i.e., exchanges that square to identity), and these generate a subgroup isomorphic to the Klein four-group, $V_{4}$. The remaining elements of $A_{4}$ include three cycles, which correspond to 2π/3 rotations and thus violate $R_{\chi }^{2}=I$. Thus, only the Klein-four $V_{4}$ subgroup remains consistent with the exchange constraint

$$R_{1}=\left[\begin{array}{cccc} 1 & 0 & 0 & 0\\ 0 & 1 & 0 & 0\\ 0 & 0 & 1 & 0\\ 0 & 0 & 0 & 1 \end{array}\right]\;R_{2}=\left[\begin{array}{cccc} 0 & 0 & 0 & 1\\ 0 & 0 & 1 & 0\\ 0 & 1 & 0 & 0\\ 1 & 0 & 0 & 0 \end{array}\right]$$ (6)

$$R_{3}=\left[\begin{array}{cccc} 0 & 1 & 0 & 0\\ 1 & 0 & 0 & 0\\ 0 & 0 & 0 & 1\\ 0 & 0 & 1 & 0 \end{array}\right]\;R_{4}=\left[\begin{array}{cccc} 0 & 0 & 1 & 0\\ 0 & 0 & 0 & 1\\ 1 & 0 & 0 & 0\\ 0 & 1 & 0 & 0 \end{array}\right]$$ (7)

One can easily see that this residual symmetry group captures the essence of chirality-preserving, spin-rotating twin exchanges in the tetrahedral configuration. Unlike independent swaps, these correlated exchanges cannot be factorized into ordinary permutations $\in S_{4}$ as in a Slater determinant. This lack of factorization produces anomalous terms in the effective single-particle Hamiltonian after contraction, analogous to anomalous pair-pair correlations in unconventional superconductivity (*54*). For the nonchiral tetrahedron, applying the same logic gives the elemental permutations

$$R_{1}^{\prime }=\left[\begin{array}{cccc} 1 & 0 & 0 & 0\\ 0 & 1 & 0 & 0\\ 0 & 0 & 1 & 0\\ 0 & 0 & 0 & 1 \end{array}\right]\;R_{2}^{\prime }=\left[\begin{array}{cccc} 0 & 1 & 0 & 0\\ 1 & 0 & 0 & 0\\ 0 & 0 & 1 & 0\\ 0 & 0 & 0 & 1 \end{array}\right]$$ (8)

$$R_{3}^{\prime }=\left[\begin{array}{cccc} 1 & 0 & 0 & 0\\ 0 & 0 & 1 & 0\\ 0 & 1 & 0 & 0\\ 0 & 0 & 0 & 1 \end{array}\right]\;R_{4}^{\prime }=\left[\begin{array}{cccc} 1 & 0 & 0 & 0\\ 0 & 1 & 0 & 0\\ 0 & 0 & 0 & 1\\ 0 & 0 & 1 & 0 \end{array}\right]$$ (9)

that are covering the full symmetric group $S_{4}$.

This analysis demonstrates that exchange statistics, dictated by symmetry, differ between chiral and nonchiral systems. In the nonchiral symmetric case, electron pairs exchange independently captured via the $R_{\chi }^{\prime }$ matrices, while, in chiral systems when one pair exchanges, the other must simultaneously exchange and this is captured via $R_{\chi }$ matrices.

### Nonlocal kernel

The twin-exchange interaction in real space is described by a nonlocal convolution, where the asymmetry seen by electron $x_{i}$ is defined relative to the others $x_{j}$. The kernel ${\hat{\Gamma }}_{R_{\chi }}$ mediates these correlations and effectively projects the many-body problem onto a single-particle description

$${\hat{\Gamma }}_{R_{\chi }}\left(\{x_{i}\}\right)=∭d^{3}x_{j}\;\Gamma_{R_{\chi }}\left({\left\{x_{i}-x_{j}\right\}}_{j\neq i}\right)\;\Psi^{\xi }\left({\left\{x_{j}\right\}}_{j\neq i}\right)$$ (10)

with $R_{\chi }$ labeling chirality-preserving twin exchanges in $V_{4}$. Fourier transformation converts the convolution into a product, making the interaction local in momentum space (*73*)

$${\tilde{\Gamma }}_{\pi }\left(\{k_{i},\gamma^{i}\}\right)\;{\tilde{\Psi }}^{\xi }\left(\{k_{i},\gamma^{i}\}\right)$$ (11)

In the long-wavelength limit ($k_{i}\to 0$), the Fourier transform of the kernel reduces to its spatial integral, leaving an effective local interaction. Physically, this corresponds to averaging the nonlocal twin exchange over distances much larger than electron separations while retaining the chirality-dependent structure encoded in the $\gamma^{i}$. In this limit, each twin exchange acts on the $\gamma^{i}$ of the participating electrons and may be written as

$${\tilde{\Gamma }}_{R_{\chi }}\left(0,\gamma^{i}\right)=K\prod_{\left\{\xi \right\}\in R_{\chi }}\gamma^{j}$$ (12)

Summing over all twin exchanges in $V_{4}$ produces the pseudoscalar structure

$$\sum_{R_{\chi }\in V_{4}}\prod_{\left\{\xi \right\}\in R_{\chi }}\gamma_{\xi }^{i}=-\text{sgn}\left(\xi \right)\;4\mathrm{iK}\gamma^{5}$$ (13)

which encodes the chirality-dependent spin interaction in the multielectron system. Note that an even/odd permutation yields a different sign on the product $\gamma^{i}\gamma^{j}\gamma^{k}\gamma^{l}=-\epsilon_{\text{ijkl}}i\gamma^{5}$ where $\epsilon_{\text{ijkl}}$ is the Levi-Civita symbol. In nonchiral systems, even and odd permutations cancel, and the anomalous terms vanish.

The additional term, $-\mathrm{iK}\gamma^{5}$, induces nontrivial ordering through twin spin-exchange interactions. Spins align according to the chirality of the system, i.e., pointing either inward toward the center of mass or outward, so that the total magnetic moment cancels out (Fig. 2C). Any alternative spin configuration on a tetrahedron would yield a net magnetic moment, which is inconsistent with experimental observations and would violate PT symmetry. Under time reversal, the spin orientation flips (inward ↔ outward), resulting in a distinct state (bottom to top row in Fig. 2C). Parity transforms the tetrahedron into its enantiomer (left to right column), without affecting the spin. Only a combined PT operation leaves the spin configuration invariant. This demonstrates that the spin-exchange interaction in the chiral four-electron system has the same PT symmetry observed in CISS, yet with a nontrivial, chirality-dependent spin texture.

Although Dirac made use of the Lorentz-invariant Clifford algebra omitting the $\gamma^{5}$ matrix in his original derivation (*55*), it has been shown that Dirac’s theory can be generalized to chiral systems where $\gamma^{5}$ also appears (*56*). This is also the only possible generalization that respects Lorentz invariance. We can now write the effective, chiral single-particle Dirac equation incorporating the above multielectron correlations effects in a complex potential as

$$\left(i\hbar \gamma^{\mu }\partial_{\mu }-\mathrm{mc}-i\frac{K}{c}\gamma^{5}\right)\;\psi =0$$ (14)

where K reflects the strength of the chiral exchange interaction and $\gamma^{5}$ ensures sensitivity to global stereochemical configuration.

### Nonrelativistic limit of Dirac equation

Assuming all other energy scales are much smaller than the electron rest mass, the derivation is restricted to the nonrelativistic limit. The chiral Dirac equation can be written in its time-independent $H\Psi_{n}=E_{n}\Psi_{n}$ for an additional electrostatic potential $\hat{V}$ in a two-spinor form with $\Psi_{n}={\left(\Psi_{n}^{L},\Psi_{n}^{S}\right)}^{T}$ with large and small components and the Hamiltonian is

$$H=\left(\begin{array}{cc} \hat{V} & c\hat{\sigma }\cdot \hat{p}\\ c\hat{\sigma }\cdot \hat{p}-2\mathrm{iK} & \hat{V}-2{\mathrm{mc}}^{2} \end{array}\right)$$ (15)

The term $\hat{\sigma }\cdot \hat{p}$ represents the inner product between the Pauli vector $\hat{\sigma }=\left(\sigma_{x},\sigma_{y},\sigma_{z}\right)$ and the momentum operator $\hat{p}=\left({\hat{p}}_{x},{\hat{p}}_{y},{\hat{p}}_{z}\right)$, expressed as $\hat{\sigma }\cdot \hat{p}=\sigma_{x}{\hat{p}}_{x}+\sigma_{y}{\hat{p}}_{y}+\sigma_{z}{\hat{p}}_{z}$. The small component can be expressed as

$$\Psi_{n}^{S}=\frac{c\hat{\sigma }\cdot \hat{p}-2\mathrm{iK}}{E_{n}-\hat{V}+2{\mathrm{mc}}^{2}}\Psi_{n}^{L}$$ (16)

Substituting into top equations yields an equation for the equation of the large equation

$$\left(\hat{V}+c\hat{\sigma }\cdot \hat{p}\frac{\hat{Q}}{2{\mathrm{mc}}^{2}}c\hat{\sigma }\cdot \hat{p}-i\hat{\sigma }\cdot \hat{p}\frac{K}{c}\hat{Q}\right)\Psi_{n}^{L}=E_{n}\Psi_{n}^{L}$$ (17)

with

$$\hat{Q}={\left(1+\frac{E_{n}-V}{2{\mathrm{mc}}^{2}}\right)}^{-1}\approx 1-\frac{E_{n}-V}{2{\mathrm{mc}}^{2}}$$ (18)

using the approximation in nonrelativistic limits yields

$$\left[\frac{{\hat{p}}^{2}}{2m}+\hat{V}-\frac{c\hat{\sigma }\cdot \hat{p}\left(E_{n}-\hat{V}\right)c\hat{\sigma }\cdot \hat{p}}{4m^{2}c^{4}}\ldots -i\hat{\sigma }\cdot \hat{p}\frac{K}{\mathrm{mc}}\left(1-\frac{E_{n}-V}{2{\mathrm{mc}}^{2}}\right)\right]\Psi_{n}^{L}=E_{n}\Psi_{n}^{L}$$ (19)

where the first two terms correspond to the usual Schrödinger equation, the third term incorporates the relativistic corrections including SOC, and the last term is the CISS term needed for the twin-pair exchange discussed above. The relevant energy scale for electrons in chiral molecules is much smaller than the rest mass of the electron of 510 keV, and, here, we neglect all relativistic corrections and replace $\alpha =\frac{K}{\mathrm{mc}}$ resulting in

$$\left(\frac{{\hat{p}}^{2}}{2m}+\hat{V}-i\alpha \hat{\sigma }\cdot \hat{p}\right)\Psi_{n}=E_{n}\Psi_{n}$$ (20)

with $\alpha =\frac{K}{\mathrm{mc}}$.

The Pauli matrices satisfy the algebra ${\left(\hat{p}\cdot \hat{\sigma }\right)}^{2}=p^{2}I$ where $p^{2}=p_{x}^{2}+p_{y}^{2}+p_{z}^{2}$ and $\hat{p}\cdot \hat{\sigma }$ has the eigenvalues ±p. Adding the $i\hat{p}\cdot \hat{\sigma }$ operator renders the Hamiltonian non-Hermitian.

The time-reversal operation $T≔-i\sigma_{y}\hat{C}$ where $\hat{C}$ is the complex conjugation operator fulfills the requirement that the linear and angular momenta are reversed $T:\hat{p}\to -\hat{p}$ and $T:\hat{\sigma }\to -\hat{\sigma }$, while the spatial coordinate is left unchanged $T:\hat{x}\to \hat{x}$ (*70*). The parity operator P inverts all spatial coordinates, thus, $P:\hat{x}\to -\hat{x}$, for the linear momenta $P:\hat{p}\to -\hat{p}$, but leaves the angular momentum, i.e., the spin orientation, unchanged $P:\hat{\sigma }\to \hat{\sigma }$ (*70*). This is because the linear momentum is a polar vector, while the angular momentum is an axial vector (*74*).

The interaction $i\hat{\sigma }$ is symmetric under $P:i\hat{\sigma }\to i\hat{\sigma }$ and $T:i\hat{\sigma }\to i\hat{\sigma }$ contrary to the CISS experimental observations, so there is no energetic difference between these four configurations. Only an operator of the form $i\hat{\sigma }\cdot \hat{p}$ breaks P and T but remains invariant under the combined PT operation

$$P:\;i\hat{\sigma }\cdot \hat{p}\;\to \;-i\hat{\sigma }\cdot \hat{p}$$ (21)

$$T:\;i\hat{\sigma }\cdot \hat{p}\;\to \;-i\hat{\sigma }\cdot \hat{p}$$ (22)

$$PT:\;i\hat{\sigma }\cdot \hat{p}\;\to \;i\hat{\sigma }\cdot \hat{p}$$ (23)

### Analytical 1D model derivations

From Eq. 2, we find that the general Hamiltonian that breaks both P and T, but not PT, symmetries is

$$H=\frac{{\hat{p}}^{2}}{2m}+\hat{V}-i\alpha \hat{p}\cdot \hat{\sigma }$$ (24)

In one dimension, the problem can be simplified. Assuming confinement in one dimension along an arbitrary *z* axis, we can write Eq. 2

$$\left(-\sigma_{0}\frac{\hbar^{2}}{2m}\frac{\partial^{2}}{\partial z^{2}}+\sigma_{z}\alpha \hbar \frac{\partial }{\partial z}+V_{0}\right)\Psi \left(x\right)=E\Psi \left(x\right)$$ (25)

This continuous particle-in-box model can be transformed into a discretized tight-binding form

$$\frac{\partial }{\partial z}\Psi \approx \frac{\Psi_{j+1}-\Psi_{j-1}}{2\Delta z}$$ (26)

and

$$\frac{\partial^{2}}{\partial z^{2}}\Psi \approx \frac{\Psi_{j+1}-2\Psi_{j}+\Psi_{j-1}}{\Delta z^{2}}$$ (27)

where one can write for the jth spin up $\Psi_{↑,j}$ and spin down $\Psi_{↓,j}$

$$\left[\begin{array}{cccccccc} a & 0 & b & 0 & 0 & 0 & \cdots & 0\\ 0 & a & 0 & c & 0 & 0 & \cdots & 0\\ c & 0 & a & 0 & b & 0 & \cdots & 0\\ 0 & b & 0 & a & 0 & c & \cdots & 0\\ \vdots & & \ddots & & \ddots & & \ddots & \vdots \\ 0 & \ldots & 0 & b & 0 & a & 0 & c\\ 0 & \ldots & 0 & 0 & c & 0 & a & 0\\ 0 & \ldots & 0 & 0 & 0 & b & 0 & a \end{array}\right]\left(\begin{array}{c} \Psi_{↑1}\\ \Psi_{↓1}\\ \Psi_{↑2}\\ \Psi_{↓2}\\ \vdots \\ \vdots \\ \Psi_{↑n}\\ \Psi_{↓n} \end{array}\right)=E\;(\begin{array}{c} \Psi_{↑1}\\ \Psi_{↓1}\\ \Psi_{↑2}\\ \Psi_{↓2}\\ \vdots \\ \vdots \\ \Psi_{↑n}\\ \Psi_{↓n} \end{array})$$ (28)

with

$$a=V_{0}+\frac{\hbar^{2}}{m\Delta z^{2}}$$ (29)

$$b=\frac{\hbar^{2}+\alpha \hbar m\Delta z}{2m\Delta z^{2}}$$ (30)

$$c=\frac{\hbar^{2}-\alpha \hbar m\Delta z}{2m\Delta z^{2}}$$ (31)

This is a diagonal banded Hamiltonian matrix, where the up and down spin components can be block diagonalized, and one sees that each of the block diagonals are Hatano-Nelson models (*58*) for the spin subspaces which are n×n tridiagonal Toeplitz matrices. The solutions to the eigenvalue problems give double-degenerated, spin-independent energies

$$E_{j}=V_{0}+\frac{\hbar^{2}}{m\Delta z^{2}}+\hbar \frac{\sqrt{\hbar^{2}-\alpha^{2}m^{2}\Delta z^{2}}}{m\Delta z^{2}}\text{cos}\frac{j\pi }{n+1}$$ (32)

Figure 3A shows the spatial distribution of the probability density of the right eigenvectors that are localized on either interface depending on spin. The right eigenvector for the spin-up state is

$$\Psi_{j,↑}\left(k\right)={\mathrm{Ae}}^{-k\text{ln}\sqrt{\frac{\hbar^{2}-\alpha \hbar m\Delta z}{\hbar^{2}+\alpha \hbar m\Delta z}}}\text{sin}\frac{\mathrm{jk}\pi }{n+1}$$ (33)

whereas the right eigenvector for the spin-down state is

$$\Psi_{j,↓}\left(k\right)={\mathrm{Ae}}^{-k\text{ln}\sqrt{\frac{\hbar^{2}+\alpha \hbar m\Delta z}{\hbar^{2}-\alpha \hbar m\Delta z}}}\text{sin}\frac{\mathrm{jk}\pi }{n+1}$$ (34)

with k=1,2,…,n. The left eigenvectors derive from the right eigenvectors as 〈ψ∣=P∣ψ〉.

Energies are real as long as ℏ>∣αΔzm∣, which is a common feature for PT systems (*44*). The use of this critical point gives an upper limit of the interaction strength of $K\approx 2\cdot 10^{2}$ eV for Δz comparable to interatomic distances. The solutions are self-consistent with the chiral spin texture discussed in Fig. 2, i.e., the resulting states are PT-symmetric where the parity operation acts on k→k−n−1 and Δz→−Δz and results in wave functions that display the opposite spin localization (Fig. 3A, left to right), i.e., the time-reversed spin texture and the original wave function are recovered by applying the time-reversal operation (Fig. 3A, top to bottom). The P operator for this particular example is a 2n×2n matrix where n is the number of sites, and it is

$$P=\left[\begin{array}{ccccc} 0 & 0 & \cdots & 0 & I\\ 0 & 0 & \cdots & I & 0\\ \vdots & \vdots & \ddots & \vdots & \vdots \\ 0 & I & \cdots & 0 & 0\\ I & 0 & \cdots & 0 & 0 \end{array}\right]$$ (35)

where I is the 2×2 identity matrix with the property $P=P^{-1}$ with eigenvalues ±1. One can see that P defined above reverses the order of the n sites but leaves the spin unchanged. The time-reversal operator is defined as $T_{n}≔-i\sigma_{2}\hat{C}⊗I_{n}$, and, thus, it reverses all the spins, but the ordering of the sites remains invariant.

The non-Hermitian skin effect refers to the accumulation of waves or particles at system boundaries due to asymmetric hopping (*48*). This directional imbalance disrupts the usual bulk-boundary correspondence of Hermitian systems, where terminating and periodic boundary conditions yield similar bulk properties. In non-Hermitian systems, however, Bloch band theory fails (*48*). This localization contrasts sharply with Hermitian systems, where, under terminating boundary conditions, a sufficiently large system smoothly transitions to the band structure with periodic boundary conditions. In non-Hermitian systems, this bulk-boundary correspondence and Bloch band theory break down (*48*). In the above non-Hermitian model with interfaces (Fig. 3A), connecting the interfaces restores periodic boundary conditions. The corresponding eigenvalues form a circle in the complex energy plane, with each spin species exhibiting two counter-propagating modes with real eigenvalues, leading to fully delocalized states (Fig. 3B). While spin-up and spin-down states could, in principle, couple weakening the skin effect, they would still localize at opposite boundaries under terminating conditions (Fig. 3A).

Spin and charge localization also arise when a structurally chiral domain is coupled to Hermitian leads, such as metallic, diamagnetic, or ferromagnetic contacts common in CISS experiments. In a simplified model, diamagnetic leads exhibit symmetric hopping and spin-independent on-site energies, while ferromagnetic leads retain symmetric hopping but introduce spin-dependent energy splitting. Figure 3 (C and D) shows the solutions of such a calculation.
